# Bithiazole Inhibitors of Phosphatidylinositol 4‐Kinase (PI4KIIIβ) as Broad‐Spectrum Antivirals Blocking the Replication of SARS‐CoV‐2, Zika Virus, and Human Rhinoviruses

**DOI:** 10.1002/cmdc.202100483

**Published:** 2021-09-07

**Authors:** Maria Grazia Martina, Ilaria Vicenti, Lisa Bauer, Emmanuele Crespan, Enrico Rango, Adele Boccuto, Noemi Olivieri, Matteo Incerti, Marleen Zwaagstra, Marika Allodi, Simona Bertoni, Elena Dreassi, Maurizio Zazzi, Frank J. M. van Kuppeveld, Giovanni Maga, Marco Radi

**Affiliations:** ^1^ Dipartimento di Scienze degli Alimenti e del Farmaco Università degli Studi di Parma Parco Area delle Scienze, 27/A 43124 Parma Italy; ^2^ Department of Medical Biotechnologies University of Siena 53100 Siena Italy; ^3^ Virology Section Infectious Disease and Immunology Division Department of Biomolecular Health Sciences Faculty of Veterinary Medicine Utrecht University Utrecht The Netherlands; ^4^ Istituto di Genetica Molecolare IGM-CNR “Luigi Luca Cavalli-Sforza” Via Abbiategrasso 207 27100 Pavia Italy; ^5^ Dipartimento Biotecnologie, Chimica e Farmacia Università degli Studi di Siena 53100 Siena Italy; ^6^ Current address: Department of Viroscience Erasmus Medical Center 3015CA Rotterdam The Netherlands; ^7^ Current address: Dipartimento di Farmacia Università degli Studi di Genova 16132 Genoa Italy

**Keywords:** Broad-spectrum antivirals, PI4KIIIb, bithiazole, rhinovirus, zika virus, SARS-CoV-2

## Abstract

Over half a century since the description of the first antiviral drug, “old” re‐emerging viruses and “new” emerging viruses still represent a serious threat to global health. Their high mutation rate and rapid selection of resistance toward common antiviral drugs, together with the increasing number of co‐infections, make the war against viruses quite challenging. Herein we report a host‐targeted approach, based on the inhibition of the lipid kinase PI4KIIIβ, as a promising strategy for inhibiting the replication of multiple viruses hijacking this protein. We show that bithiazole inhibitors of PI4KIIIβ block the replication of human rhinoviruses (hRV), Zika virus (ZIKV) and SARS‐CoV‐2 at low micromolar and sub‐micromolar concentrations. However, while the anti‐hRV/ZIKV activity can be directly linked to PI4KIIIβ inhibition, the role of PI4KIIIβ in SARS‐CoV‐2 entry/replication is debated.

## Graphical abstract

**Host targeting** is a promising approach for the development of broad‐spectrum antiviral agents (BSAAs) endowed with a high genetic barrier to resistance and efficacy against viral mutants resistant to conventional antiviral drugs. We show that bithiazole inhibitors of the host lipid kinase PI4KIIIβ block the replication of human rhinoviruses (hRV), Zika virus (ZIKV) and SARS‐CoV‐2 at low micromolar and sub‐micromolar concentrations. Moreover, inhibition of SARS‐CoV‐2 entry seems to be connected with an additional unknown target.

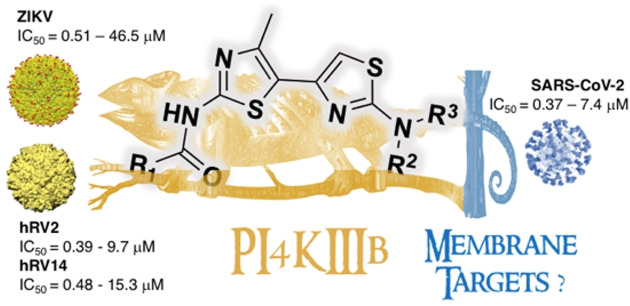

Most antiviral drugs that are currently on the market target viral proteins, typically viral enzymes and their catalytic domains (e. g. HIV reverse transcriptase and protease, or influenza virus neuraminidase). Despite latest generation of these drugs are highly selective for viral proteins and display low toxicity for the host, older drugs still in clinical use are affected by substantial acute and/or long‐term toxicity, thus questioning the general assumption on their high safety. Most importantly, viruses (especially those with a RNA genome) have a high mutation rate that allows to easily select drug resistant strains by alteration of target viral proteins, thus jeopardizing the long‐term clinical use of such compounds. Considering the limited antiviral arsenal currently available, antiviral drug‐resistance is a major concern particularly for medically important persistent viruses (e. g. HIV, Hepatitis B and C virus). In addition, “old” re‐emerging viruses (e. g. chikungunya virus, ebola virus, dengue virus) and new emerging viruses (e. g. SARS‐CoV, MERS‐CoV and other related viruses) represent a serious threat for global public health and prompt intervention is needed to develop new antiviral drugs. Since host proteins are less prone to mutate, antiviral research has recently started to explore drugs targeting cellular proteins essential for virus replication with the aim of minimizing emergent drug resistance.[^1^, ^2^] Host targeting antivirals represent therefore a promising approach for the treatment of rapidly spreading and mutation‐prone viruses such as the new severe acute respiratory syndrome coronavirus‐2 (SARS‐CoV‐2) responsible for the current pandemic.

Viruses are obligate intracellular parasites that heavily depend on the host cell. Therefore, most viruses rely on host protein kinases to catalyse essential phosphorylation steps needed for virus replication. Since only very few viruses (rotaviruses, poxviruses, herpesviruses) encode for these enzymes on their own, most usually they hijack host kinases and other proteins of the host machinery.^[3]^ One fundamental difference between viruses and eukaryotic cells is that the life cycle of different viruses can depend on the activity of specific kinases, while kinase‐dependent metabolic pathways in cells are often redundant so that one kinase could be substituted by another one. Thus, there is a therapeutic window of opportunity, where timely administration of a kinase‐targeting drug will block the viral replication without causing excessive toxicity to uninfected cells. In addition, antiviral drugs targeting host kinases are expected to: *i)* retain activity against viral mutants resistant to conventional antiviral drugs; *ii)* minimize drug escape, since host proteins are genetically stable; *iii)* be active against viruses belonging to different virus families that rely on the same kinase, resulting in broad‐spectrum antiviral agents (BSAAs) which are also useful for outbreaks of newly emerged viruses. So far, a number of host kinases have been identified as promising targets for the development or repurposing of kinase inhibitors as antiviral agents: as reported in a recently published review, kinase inhibitors block the replication of many viruses by interfering with different steps of the viral replication cycle.^[4]^ In particular, phosphatidylinositol kinases (PIKs) play a key role in the formation of replication organelles (ROs), which are the central hub for positive‐strand RNA virus (+RNA virus) replication and protect the viral genome from host defenses.[^5^, ^6^, ^7^] Among the different lipid kinases responsible for the conversion of phosphatidylinositols (PI) into phosphoinositides (PPIns) needed for cellular processes, the PI3K, PI4K, PIP5KI, and PIKfyve families are those exploited by several viruses (e. g. SARS, MERS, EBOV, HCV, enteroviruses and rhinoviruses, HIV‐1) for entry or replication in cells.^[8]^ Several viruses recruit a specific set of proteins to the ROs: as an example, enteroviruses and rhinoviruses hijack the phosphatidylinositol 4‐kinase type IIIβ (PI4KIIIβ) to enrich for phosphatidylinositol‐4‐phosphate (PI4P) at their ROs to facilitate viral replication.^[9]^ PI4KIIIβ also plays a key role in the replication of some members of different virus families: *i)* since PI4P is also enriched at Zika virus (ZIKV) ROs, its replication was also inhibited by PI4KIIIβ inhibitors;^[10]^ *ii)* SARS‐CoV pseudovirus proved to be dependent on PI4KIIIβ for spike‐mediated entry into the cell whereas PI4KIIIα had no effect and PI3KR1 played an opposite role by regulating PI4P concentration in opposite directions.^[11]^ Specific inhibition of PI4KIIIβ vs PI3KR1 seems therefore a promising approach for the inhibition of SARS‐CoV entry. In addition, PI4KIIIβ has been also proposed as potential target against SARS‐CoV‐2 in a recent proteomic/chemoinformatic analysis focused on the identification of druggable host factors for SARS‐CoV‐2.^[12]^ As partial confirmation of the last assumption, Yang H. et al. have shown that SARS‐CoV‐2 pseudoviruses enter the cell via pH‐dependent endocytosis and that PI4KIIIβ is required to enter the cell in a caveolae‐independent manner.^[13]^ However, to the best of our knowledge, no definitive reports on the effect of PI4KIIIβ inhibitors against SARS‐CoV‐2 replication in infected cells have been published so far. Small‐molecule inhibitors of PI4KIIIβ (Figure 1) can block virus replication without any major effect on cell viability since the PI4P amount produced by other PI4Ks could support cell trafficking and signalling but is not sufficient to properly sustain viral RNA synthesis, thus creating a therapeutic window for inhibition of virus replication without affecting cell viability.^[14]^ Even if serious doubts on the *in vivo* safety of PI4KIIIβ‐targeting antivirals have been reported for some candidates^[15]^ it is plausible that the *in vivo* safety of PI4KIIIβ inhibitors could be chemotype‐specific and possibly improved by combination therapies or multi‐target agents with reduced on‐target related toxicity.[^16^, ^17^] As a continuation of our ongoing efforts in the discovery of new broad‐spectrum antiviral agents,[^18^, ^19^] we report herein a preliminary study aimed at exploring the broad‐spectrum antiviral efficacy of bithiazole‐based PI4KIIIβ inhibitors. Target compounds were thus designed, synthesized and evaluated against different viruses to establish the BSA potential of the bithiazole chemotype.

**Figure 1:**
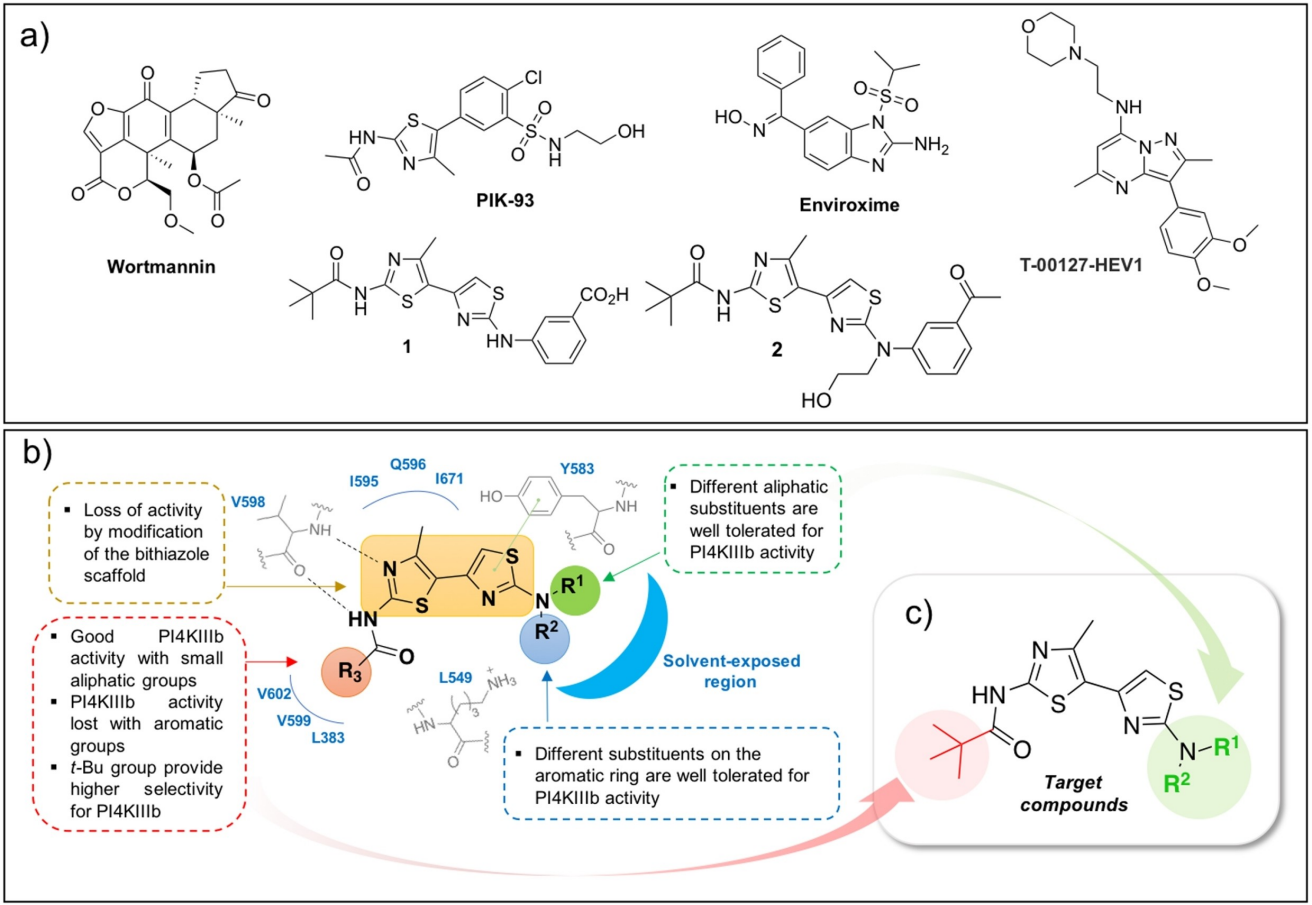
a) Representative PI4KIIIβ inhibitors endowed with broad‐spectrum antiviral activity. b) SAR of PI4KIIIβ‐targeting bithiazole antivirals. c) Target compounds of the present work.

In the search for new multi‐target agents for treatment of cystic fibrosis and related viral infections, we have recently discovered a family of bithiazole derivatives that proved to inhibit the replication of several enteroviruses by targeting the host PI4KIIIβ kinase.[^20^, ^21^] Among the synthesized derivatives, compounds **1** and **2** (Figure 1a) represent the most promising candidates: at low micromolar concentration, both compounds inhibited the enzymatic activity of PI4KIIIβ and the replication of Enterovirus‐A71 (EV–A71), coxsackievirus B3 (CVB3), human rhinovirus 2 (hRV2) and human rhinovirus 14 (hRV14) in infected cells. Compound **2** was also well tolerated by C57BL/6 mice with no sign of acute toxicity and histological alterations in key biodistribution organs up to 180 mg/kg. Based on the previously published results for this family of compounds, clear structure‐activity‐relationships (SAR) for the inhibition of PI4KIIIβ can be drawn (Figure 1b): *i)* the bithiazole core is fundamental for the activity; *ii)* R_3_ bulkiness cannot exceed that of a *t*‐Bu; *iii)* different R_1_ and R_2_ substituents allow to retain anti‐PI4KIIIβ activity and point toward a solvent‐exposed region. One problem with these compounds is the low water solubility and thus, we decided to prepare a few additional bithiazole derivatives bearing less lipophilic moieties in R_1_/R_2_ to have a small set of compounds with different lipophilicity to study their antiviral effect (Figure 1c). All target compounds should be characterized by a *t*‐Bu group in R_3_, found responsible for the high selectivity toward PI4KIIIβ, and different aliphatic/polar substituents in R_1_/R_2_. The desired bithiazoles **4 a**–**d** were simply prepared by reacting the key intermediate **3**, obtained in three steps as previously reported,[^20^, ^21^] with substituted thioureas **9 a**–**b** under reflux in ethanol. The latter compounds were, in turn, obtained from the corresponding amines via a two steps protocol consisting in a reaction with benzoyl isothiocyanate followed by deprotection with sodium methoxide. The sole non‐commercial amine **7 d** was prepared by reductive amination between the cyclohexanone **5** and the alcohol **6** (Scheme 1). Synthesized compounds were initially evaluated for their effect on the enzymatic activity of two different lipid kinases: PI4KIIIβ vs PI3KR1. As reported in Table 1, compounds **4 a**–**d** did not show any effect on PI3KR1 but PI4KIIIβ activity was inhibited at low micromolar concentrations as previously observed for compounds **1** and **2** (Table 1). The whole set of compounds (**1**, **2**, **4 a**–**d**) is therefore characterized by a similar effect against the lipid kinases of interest and by a wide lipophilicity range according to the predicted LogP values (cLogP, Table 2). Next, we evaluated the antiviral efficacy of the PI4KIIIβ inhibitors against viruses from different virus families. As positive controls for virus replication inhibitors, Remdesivir (RMD), Camostat (CMT), Sofosbuivir (SOF) and the PI4KIIIβ inhibitor BF738735 (Table 1) were used. As expected from previous screening of **1** and **2** against hRV (Table 1, entries 1–2) and from PI4KIIIβ IC_50_ data, the new compounds **4 a**–**d** inhibited hRV2 and hRV14 replication at low micromolar concentrations, with a potency comparable to that of the reference BF738735.^[22]^ To determine the effect of **4 a**–**d** on PI4P synthesis, a PI4P stain was performed: cells were treated for 1 h with 10 μM of each compound, stained for PI4P and imaged with a fluorescence microscope. Treated cells showed less PI4P staining compared to the negative control DMSO (Figure S1, Supporting Information).

**Scheme 1:**
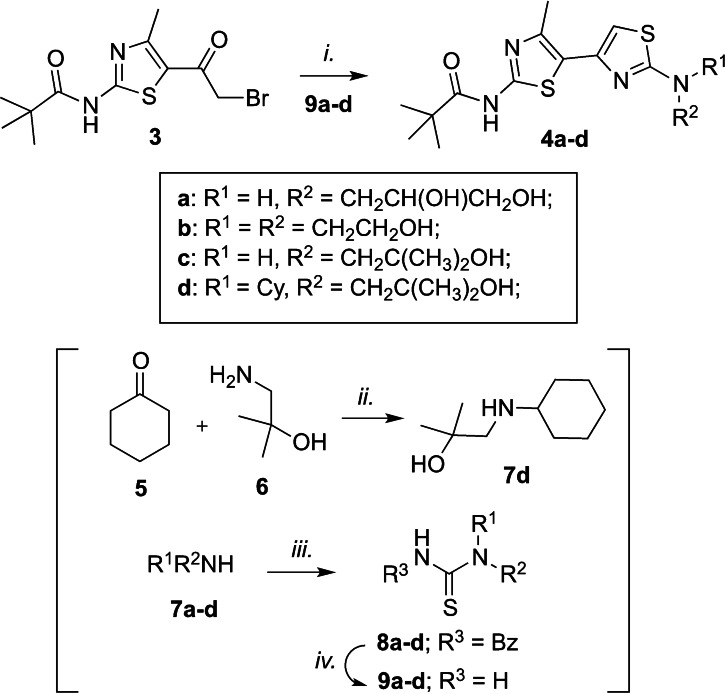
Reagents and conditions: *i*. EtOH, reflux, 1–2 h; *ii*. NaBH_4_, EtOH, 0–25 °C, 4 h; *iii*. Benzoyl isothiocyanate, DCM dry, 25 °C, 1–2 h; *iv*. MeOH dry NaOMe 25 °C, 2–19 h;

**Table 1:** Activity of bithiazole derivatives **1**, **2** and **4 a**–**d** against selected lipid kinases and RNA viruses.

Entry	ID	PI4KIIIβ	PI3KR1	SARS‐CoV‐2		ZIKV		hRV2	hRV14		Human Lymphocytes
				Calu‐3		Huh7		HeLa			
		IC_50_ [μM][^a]^		IC_50_ [μM]^[b]^	CC_50_ [μM]^[c]^	IC_50_ [μM]	CC_50_ [μM]	IC_50_ (μM)	IC_50_ [μM]	CC_50_ [μM]	Viability^[d]^ @50uM
1	**1** ^[e]^	0.09±0.01	NA^[f]^	7.45±2.47	45	13.79±4.08	110	9.70	15.30	36	94.1
2	**2** ^[e]^	2.10±0.17	NA	3.95±1.48	40	1.00±0.10	16	6.10	>9.10	17	94.5
3	**4 a**	0.29±0.08	NA	NA	>100	46.50±2.96	>200	1.77±0.62	2.32±1.54	>30	96.0
4	**4 b**	0.24±0.05	NA	NA	>100	5.00±1.20	125	1.37±1.31	2.07±1.45	>30	92.6
5	**4 c**	0.89±0.20	NA	NA	>100	0.83±0.31	37	0.39 ±0.01	0.48±0.14	>30	98.3
6	**4 d**	1.97±0.79	NA	1.57±0.38 (0.37±0.08)	17	0.51±0.05	4	0.90±0.72	1.40±1.20	23.8±1.7	94.8
7	**RMD**	ND^[g]^	ND	0.11±0.04	97	ND	ND	ND	ND	ND	ND
8	**CMT**	ND	ND	0.82±0.32 (0.04±0.01)	200	ND	ND	ND	ND	ND	ND
9	**SOF**	ND	ND	ND	ND	2.70±0.50	200	ND	ND	ND	ND
10	**BF738735**	ND	ND	ND	ND	ND	ND	0.39±0.36	0.59±0.28	>30	ND

[a] Values are the mean of at least three independent experiments; [b] IC50: half‐maximal inhibitory concentration calculated with the DYRA protocol ±standard deviation (SD); data for active compounds under ENTRY‐DYRA conditions are reported in parenthesis; [c] CC_50_: half‐maximal cytotoxic concentration; [d] Expressed as percentage of viable human lymphocytes with respect to vehicle (DMSO 0.5 %); [e] hRV02 and hRV14 data have been taken from Ref. [21]; [f] NA: Not active; [g] ND: Not determined.

**Table 2:** ADME properties of bithiazole derivatives **1**, **2** and **4 a**–**d**.

Cpd	cLogP	Aqueous Solubility^[a]^ [μg/mL]	P_app_ 10^−6^ cm/sec (% MR^[b]^)	Plasma Stability^[c]^ [%]	Stability in Medium/Serum^[d]^ [%]	
					24 h	48 h
**1**	4.96	0.21	0.052 (<0.1)	99.1	96.4	93.1
**2**	4.39	0.05*	1,1* (<0.1)	>99*	98.5	90.4
**4 a**	2.01	5.26	0.008 (<0.1)	99.4	99.0	97.5
**4 b**	2.59	0.28	0.021 (0.94)	90.2	91.4	84.9
**4 c**	3.34	0.32	0.323 (<0.1)	92.3	77	67.3
**4 d**	5.77	0.01	11.729 (18.05)	92.4	93.3	85.7

*Previously published in ref. [21]. [a] In buffer solution at pH 7.4 (25 mM HEPES, 140 mM NaCl). [b] Membrane Retention (%MR) expressed as percentage of compound unable to reach the acceptor compartment. [c] After 24 h of incubation in human plasma solution. [d] Incubation in EMEM, 2 mM L‐glut, 1 % FBS, 1 % Pen/Strep.

Target compounds were then tested against ZIKV, whose replication is known to be attenuated by PI4KIIIβ inhibitors such as IN‐9.^[9]^ As reported in Table 1, all compounds inhibited ZIKV replication in Huh7 cells with IC_50_ comparable (**2**, **4 a**) or even five‐times lower (**4 c**, **4 d**) than that of the reference compound SOF.^[23]^ Next, we evaluated the efficacy of our PI4KIIIβ inhibitors on SARS‐CoV‐2 replication in pulmonary Calu‐3 cells, which better mimic lung infection, following two experimental protocols: *i)* the direct yield reduction assay (DYRA), which foresees the incubation of infected cells and inhibitors for 72 hours; *ii)* a variation of the DYRA protocol (ENTRY‐DYRA) tailored for the evaluation of entry inhibitors, which foresees the incubation of cells and compounds for 1 h, followed by virus adsorption, removal of inhibitors/viruses and incubation for 72 hours (see Supporting Information for details). Compounds **1**, **2** and **4 d** inhibited the replication of SARS‐CoV‐2 at micromolar or sub‐micromolar concentrations while **4 a**–**c** did not show any significant inhibition. Compound **4 d** and the SARS‐CoV‐2 entry inhibitor CMT^[24]^ were the only molecules effective under ENTRY‐DYRA conditions, indicating an inhibitory effect in the early phases of SARS‐CoV‐2 cell entry. As in the case of CMT, the effect of **4 d** in the entry phase of SARS‐CoV‐2 is further supported by the lower efficacy of both compounds under DYRA conditions (Table 1). Despite **4 d** did show suboptimal selectivity index (SI) against ZIKV (SI=8) and SARS‐CoV‐2 under DYRA conditions (SI=11), its antiviral effect was not related to the cytotoxic effect since cell lines showed a viability higher than 95 % when exposed to the highest concentration tested in the antiviral assay. To better understand if the borderline cytotoxicity of **4 d** and other analogues could be connected to the specific immortalized cell line used for virus amplification, primary human lymphocytes were incubated with 50 μM concentration of each compound. As reported in Table 1, a cell viability higher that 90 % was found in all cases, possibly suggesting a lower toxicity profile in primary cells that may support further investigations of this chemotype. Finally, we also analysed a few *in vitro* ADME properties: aqueous solubility, membrane apparent permeability (P_app_), stability in human plasma, and stability in medium/serum at different times (Table 2). Compounds’ lipophilicity (cLogP) was theoretically calculated and proved to be in line with the experimental aqueous solubility: only in the case of compound **1**, the solubility was slightly higher than expected, probably due to the hydrolysis of the carboxy group. All compounds showed high stability in plasma, while in medium/serum only compound **4 c** showed some time‐dependent decomposition. Interesting results were obtained by running the membrane apparent permeability with PAMPA assay under conditions that simulated the ENTRY‐DYRA protocol: among all compounds, **4 d** was the only one showing a good membrane permeability and a high membrane retention after 5 hours incubation at room temperature.

Overall, the biological results herein presented highlight the broad‐spectrum antiviral potential of this family of bithiazole derivatives, which proved to inhibit the enzymatic activity of PI4KIIIβ (with no effect on PI3KR1), to reduce intracellular PI4P levels and thus, inhibit replication of viruses belonging to different families at low micromolar concentration. However, while the anti‐ZIKV and anti‐rhinovirus activity may be in line with the effect on PI4KIIIβ, a few considerations may still question the observed role of PI4KIIIβ in SARS‐CoV‐2 replication: *i)* sub‐micromolar PI4KIIIβ inhibitors **4 b**–**c** did not show any effect on SARS‐CoV‐2 replication under both experimental protocols; *ii)* the most potent PI4KIIIβ inhibitor **1** and compound **2** inhibited SARS‐CoV‐2 replication only under DYRA conditions with no effect on the entry phase; *iii)* the less potent PI4KIIIβ inhibitor **4 d** inhibited SARS‐CoV‐2 replication under both experimental protocols with IC_50_s one order of magnitude higher then CMT (ENTRY‐DYRA protocol) and RMD (DYRA protocol). This data suggests that the effect of compounds **1**, **2** and **4 d** on SARS‐CoV‐2 replication is not connected to PI4KIIIβ inhibition and is possibly due to the inhibition of a different unknown target. The inhibition of SARS‐CoV‐2 entry observed only with compound **4 d** could somehow be connected with its high lipophilicity and phospholipids affinity, as demonstrated by the substantial membrane retention after 5 hours incubation. It is thus possible that the anti‐SARS‐CoV‐2 activity of compounds **1** and **2** could result from a prolonged membrane interaction during the 72 hours incubation (DYRA protocol) and/or to a lower affinity for the unknown target inhibited also by **4 d**.

In summary, the bithiazole chemotype can be considered a promising scaffold for the development of BSAAs by targeting the host kinase machinery (PI4KIIIβ) or even additional targets as it could be the case herein shown with SARS‐CoV‐2. As we have previously described, it is possible to tweak the bithiazole chemotype to obtain multitarget derivatives acting simultaneously on different targets involved in different diseases (e. g. cystic fibrosis and related viral infections).[^20^, ^21^] The data reported in this paper may therefore guide the design of tailored bithiazoles with an improved broad‐spectrum antiviral activity by simultaneous inhibition of multiple targets involved in the replication of multiple viruses. Further studies on this class of BSAAs are currently ongoing to better understand their mechanism of action and antiviral potential. Additional results will be published in due course.

## Experimental Section

Procedures for compounds synthesis and biological evaluation are reported in the Supporting Information.

## Conflict of interest

The authors declare no conflict of interest.

## Supporting information

As a service to our authors and readers, this journal provides supporting information supplied by the authors. Such materials are peer reviewed and may be re‐organized for online delivery, but are not copy‐edited or typeset. Technical support issues arising from supporting information (other than missing files) should be addressed to the authors.

Supporting Information
