# Supplementary material for: Bithiazole Inhibitors of Phosphatidylinositol 4‐Kinase (PI4KIIIβ) as Broad‐Spectrum Antivirals Blocking the Replication of SARS‐CoV‐2, Zika Virus, and Human Rhinoviruses
Source: ChemMedChem. 2021 Sep 7;16(23):3548–52. doi: 10.1002/cmdc.202100483 (PMC8427023; doi:10.1002/cmdc.202100483)
Supplement: Supplementary file 1 — Supporting Information [file CMDC-16-3548-s001.pdf]

ChemMedChem

Supporting Information

Bithiazole Inhibitors of Phosphatidylinositol 4-Kinase (PI4KIIIβ) as Broad-Spectrum Antivirals Blocking the Replication of SARS-CoV-2, Zika Virus, and Human Rhinoviruses

Maria Grazia Martina, Ilaria Vicenti, Lisa Bauer, Emmanuele Crespan, Enrico Rango, Adele Boccuto, Noemi Olivieri, Matteo Incerti, Marleen Zwaagstra, Marika Allodi, Simona Bertoni, Elena Dreassi, Maurizio Zazzi, Frank J. M. van Kuppeveld, Giovanni Maga, and Marco Radi*

TABLE OF CONTENTS:

1)	**CHEMISTRY**	Pag S2
-	General	Pag S2-S3
-	Compounds’ synthesis and characterization	Pag S3-S5
-	Water solubility	Pag S5
-	PAMPA assay	Pag S6
-	Stability tests	Pag S6
2)	**BIOLOGY**	Pag S7
-	ZIKV/SARS-CoV-2 testing	Pag S7-S8
-	HRV-02 / HRV-14 testing	Pag S8
-	PI(4)P lipid staining	Pag S9
-	Supplementary Figure S1:	
	Effect of compounds **4a-4d** on intracellular PI4P levels.	Pag S10
-	Viability assays in human lymphocytes	Pag S11
-	In vitro kinase inhibition assays	Pag S11
-	References	Pag S12

MATERIAL AND METHODS

1) Chemistry

General. All commercially available chemicals were purchased from Merck or Fluorochem and, unless otherwise noted, used without any previous purification. Solvents used for work-up and purification procedures were of technical grade. TLC was carried out using Merck TLC plates (silica gel on Al foils, SUPELCO Analytical). Where indicated, products were purified by silica gel flash chromatography on columns packed with Merck Geduran Si 60 (40-63 µm). ^{1}H and ^{13}C NMR spectra were recorded on BRUKER AVANCE 300 MHz and BRUKER AVANCE 400 MHz spectrometers. Chemical shifts (δ scale) are reported in parts per million relative to TMS. ^{1}H-NMR spectra are reported in this order: multiplicity and number of protons; signals were characterized as: *s* (singlet), *d* (doublet), *dd* (doublet of doublets), *ddd* (doublet of doublet of doublets), *t* (triplet), *m* (multiplet), *bs* (broad signal). Low resolution mass spectrometry measurements were performed on Quattro micro API tandem mass spectrometer (Waters, Milford, MA, USA) equipped with an external APCI or ESI ion source. ESI-mass spectra are reported in the form of (*m/z*). Melting points were taken using a Gallenkamp melting point apparatus and were uncorrected. Elemental analyses were performed on a ThermoQuest (Italia) FlashEA 1112 Elemental Analyzer. All final compounds showed chemical purity ≥95% as determined by elemental analysis data for C, H, and N (within 0.4% of the theoretical values).

Microwave Irradiation Experiments. Microwave reactions were conducted using a CEM Discover Synthesis Unit (CEM Corp., Matthews, NC). The machine consists of a continuous focused microwave power delivery system with an operator-selectable power output from 0 to 300 W. The temperature inside the reaction vessel was monitored using a calibrated infrared temperature control mounted under the reaction vessel. All experiments were performed using a stirring option whereby the reaction mixtures were stirred by means of a rotating magnetic plate located below the floor of the microwave cavity and a Teflon-coated magnetic stir bar in the vessel.

ADME analysis. UV/LC-MS analyses of water solubility (for compounds **1**, **4a**, **4b** and **4c**), PAMPA, and Stability Tests (in human plasma and medium/serum solution) were performed by using Agilent 1100 LC/MSD VL system (G1946C) (Agilent Technologies, Palo Alto, CA) constituted by a vacuum solvent degassing unit, a binary high-pressure gradient pump, an 1100 series UV detector, and an 1100 MSD model VL benchtop mass spectrometer. MSD single-quadrupole instrument was equipped with the orthogonal spray API-ES (Agilent Technologies, Palo Alto, CA). The pressure of the nebulizing gas and the flow of the drying gas (nitrogen used for both) were set at 40 psi, 9 L/min, respectively. The capillary voltage, the fragmentor voltage, and the vaporization temperature were 3000 V, 70 V, and 350 °C, respectively. MSD was used in the positive and negative ion mode. Spectra were acquired over the scan range m/z 50–1500 using a step size of 0.1. Chromatographic separation was obtained using a Phenomenex Kinetex C18-100Å column (150 x 4.6 mm, 5 µm

particle size) at room temperature and gradient elution with a binary solution (eluent A: H_2O, eluent B: ACN; both eluents were acidified with formic acid 0.1% v/v). The analysis started with 5% of B (from t = 0 to t = 3 min), then B was increased to 95% (from t = 3 to t = 12 min), then kept at 95% (from t = 12 to t = 18 min) and finally return to 5% of eluent B in 2.0 min. The flow rate was 0.6 mL/min and injection volumes were 10 μL. UV detection was monitored at 254 nm. Quantification of the single compound was made by comparison with appropriate calibration curves.

The water solubility of compound **4d** was determined using an LC-MS/MS system consisting of a Varian apparatus (Varian Inc) including a vacuum solvent degassing unit, two pumps (212-LC), an MSD triple quadrupole mass spectrometer (Mod. 320-LC) with ES interface and Varian MS Workstation System Control Vers. 6.9. Chromatographic separation was obtained using a Phenomenex Kinetex C18-100Å column (20 x 2.1 mm, 2.6 μm particle size) and gradient elution with a binary solution (eluent A: H_2O, eluent B: ACN; both eluents were acidified with formic acid 0.1% v/v). The analysis started with 5% of B (from t = 0 to t = 1 min), then B was increased to 95% (from t = 1 to t = 8 min), then kept at 95% (from t = 8 to t = 10 min) and finally return to 5% of eluent B in 2.0 min. The flow rate was 0.2 mL/min and injection volumes were 5 μL. The instrument operated in positive mode and parameters were: detector 1850 V, drying gas pressure 25.0 psi, desolvation temperature 300.0 °C, nebulizing gas 45.0 psi, needle 5000 V and shield 600 V. Nitrogen was used as nebulizer gas and drying gas. Collision induced fragmentation was performed using Argon as the collision gas at a pressure of 1.8 mTorr in the collision cell. The capillary voltage was optimized at 104 V and the quantification of **4d** was performed using a mass transition of 450.66 → 433.0 with a collision energy of 22.0 V. Quantification was made by comparison with appropriate calibration curve.

COMPOUNDS' SYNTHESIS AND CHARACTERIZATION

General Procedure for the Synthesis of 1-(cyclohexylamino)-2-methylpropan-2-ol (7d): To a solution of 1-amino-2-methylpropanol (200 mg; 2.25 mmol) in ethanol (3.39 mL), cyclohexanone (465 μL; 4.49 mmol) was added and the resulting mixture was stirred at 0-10°C for 10 minutes under N_2 atmosphere. $NaBH_4$ (160 mg; 4.28 mmol) was added at 10°C, then the mixture was stirred at room temperature for 4 hours. After that time, the reaction mixture was quenched by adding water, then filtered through a Celite pad and finally evaporated under reduced pressure. The resulting residue was dissolved in 1N HCl and extracted once with ethyl acetate. The pH of the aqueous layer was adjusted to 8 using saturated sodium bicarbonate solution and then washed six times with ethyl acetate and twice with chloroform. The organic phase was dried over sodium sulphate and concentrated under vacuum to obtain the pure product **7d**.

Yield: 64%. ^{1}H-NMR ($CDCl_3$ 400 MHz): δ 1.01 -1.10 (m, 2H), 1.16 (s, 6H), 1.20 - 1.31 (m, 3H), 1.60 - 1.65 (m; 1H), 1.72 - 1.76 (m, 2H), 1.89 - 1.93 (m, 2H), 2.37 - 2.44 (m, 1H), 2.55 (s, 2H).

General Procedure for the Synthesis of compounds (8a-d): Benzoyl isothiocyanate (302 µL; 2.25 mmol) was added dropwise to a solution of the proper amine (**7a-d**) (2.25 mmol) in dichloromethane (7.29 mL). The mixture was stirred at room temperature for 1-2 h and then evaporated under reduced pressure. The resulting crude was purified by flash chromatography using the proper eluent: **8a** (CH_2Cl_2/MeOH 99:1-98:2); **8b** (CH_2Cl_2/MeOH 98:2-97:3); **8c** (petroleum ether/ethyl acetate 85:15-7:3). **8d** was used in the next step without further purification.

N-((2,3-dihydroxypropyl)carbamothioyl)benzamide (8a): Yield: 44%; ^{1}H-NMR ($CDCl_3$ 400 MHz): δ 3.65-3.69 (m, 1H); 3.76-3.86 (m, 2H); 3.98-4.08 (m, 2H); 7.52 (t, 2H, *J* = 8 Hz); 7.63 (t, 1H, *J* = 8 Hz); 7.85 (m, 2H); 9.17 (s, 1H); 11.02 (m, 1H).

N-(bis(2-hydroxyethyl)carbamothioyl)benzamide (8b): Yield: 87%; ^{1}H-NMR (MeOD 400 MHz): δ 3.89 (s, 4H), 4.00 (m, 2H); 4.10 (m, 2H); 7.50-7.53 (m, 2H); 7.60-7.62 (td, 1H, *J* = 4 Hz, *J* = 8 Hz); 7.91-7.93 (m, 2H).

N-((2-hydroxy-2-methylpropyl)carbamothioyl)benzamide (8c): Yield: 77%; MS (ESI) $[M+H]^+$ = 253,33 m/z; ^{1}H-NMR (DMSO-d_6 400 MHz): δ 1.17 (s, 6H); 3.55 (d, 2H, *J* = 4 Hz); 4.88 (s, 1H); 7.48-7.52 (m, 2H); 7.64 (td, 1H, *J* = 4 Hz, *J* = 8 Hz); 7.91-7.94 (m, 2H); 11.11 (t, 1H, *J* = 8 Hz); 11.31 (s, 1H).

N-(cyclohexyl(2-hydroxy-2-methylpropyl)carbamothioyl)benzamide (8d): Yield: 95%; ^{1}H-NMR ($CDCl_3$ 400 MHz): δ 1.21-1.27 (m, 2H); 1.32 (s, 6H); 1.40-1.46 (m, 2H); 1.72 (s, 3H); 1.80-1.84 (m, 2H); 1.91-1.94 (m, 2H); 3.37-3.39 (m, 2H); 7.45-7.50 (m, 2H); 7.54-7.58 (m, 1H); 7.82-7.86 (m, 2H).

General Procedure for the Synthesis of compounds (9a-d): Sodium (209 mg; 4.76 mmol) was added to dry MeOH (10 mL) under argon atmosphere and stirred until a clear solution of sodium metoxide was obtained. The proper intermediate **8a-d** (1.59 mmol) was added portionwise to the sodium metoxide solution. The resulting mixture was stirred under argon atmosphere at room temperature for 2h for **8a-c**, 19h for **8d**. The solvent was finally evaporated under reduced pressure and the resulting crude purified by flash chromatography using the proper eluent: **9a** (CH_2Cl_2/MeOH 93:7); **9b** (CH_2Cl_2/MeOH 97:3-96:4); **9c (**CH_2Cl_2/MeOH 93:7-95:5); **9d (**CH_2Cl_2/MeOH 99,5:0,5-98:2).

1-(2,3-dihydroxypropyl)thiourea (9a): Yield: 80%; ^{1}H-NMR (DMSO-d_6 300 MHz): *δ* 3.17 (s, 1H); 3.25-3.55 (m; 4H).

1,1-bis(2-hydroxyethyl)thiourea (9b): Yield: 30%; MS (ESI) $[M+H]^+$ = 165,13 m/z; ^{1}H-NMR (DMSO-d_6 300 MHz): *δ* 3.58-3.69 (m, 8H); 4.87 (bs, 2H); 7.21 (s, 2H).

1-(2-hydroxy-2-methylpropyl)thiourea (9c): Yield: 90%; MS (ESI) $[M+H]^+$: 149,06 m/z; ^{1}H-NMR (DMSO-d_6 300 MHz): δ 1.08 (s, 6H); 4.58 (m, 2H); 7.05 (s, 2H).

1-cyclohexyl-1-(2-hydroxy-2-methylpropyl)thiourea (9d): Yield: 33%; MS (ESI) $[M+H]^+$ = 231,19 m/z; ^{1}H-NMR ($CDCl_3$ 400 MHz): 1.02-1.12 (m, 1H); 1.21-1.24 (m, 2H); 1.32 (s, 6H); 1.40-1.50 (m, 2H); 1.67-1.72 (m, 4H); 1.80-1.83 (m, 2H); 1.91-1.94 (m, 2H); 2.65 (bs, 1H).

General procedure for the synthesis of compounds (4a-d): A solution of N-(5-(2-bromoacetyl)-4-methylthiazol-2-yl)pivalamide (86 mg; 0.27 mmol) and the proper thiourea (**9a-d**) in ethanol (1.88 mL) was stirred under reflux for 1 hour (compounds **4a**, **4b**) or 2 hours (compounds **4c** and **4d)**. After evaporation of the solvent, saturated aqueous $NaHCO_3$ was added and the mixture was extracted three times with ethyl acetate. The combined organic phases were dried over Na_2SO_4 and concentrated under vacuum. The crude was purified by flash chromatography using the proper eluent: **4a** (CH_2Cl_2/MeOH 97:3); **4b-d** (CH_2Cl_2/MeOH 98:2).

N-(2'-((2,3-dihydroxypropyl)amino)-4-methyl-[5,5'-bithiazol]-2-yl)pivalamide 4a: Yield: 67%; MS (ESI) $[M+H]^+$ = 371,05 m/z; ^{1}H-NMR (DMSO-d_6 300 MHz): δ 1.23 (s, 9H); 2.51 (s, 3H); 3.37 (m, 1H); 3.68 (m, 1H); 4.63 (t, 1H, J = 8 Hz); 4.88 (d, 1H, J = 8 Hz); 6.59 (s, 1H); 7.68 (t, 1H, J = 4 Hz); 11.45 (s, 1H); ^{13}C-NMR (DMSO-d_6, 75 MHz): δ 17.34; 27.08; 39.18; 48.35; 64.14; 70.67; 100.88; 121.09; 142.55, 143.03; 156.00; 168.81; 176.87.

N-(2'-(bis(2-hydroxyethyl)amino)-4-methyl-[5,5'-bithiazol]-2-yl)pivalamide 4b: Yield: 62%; MS (ESI) $[M+H]^+$ = 385,33 m/z; ^{1}H-NMR (DMSO-d_6 300 MHz): δ 1.23 (s, 9H); 2.45 (s, 3H); 3.54 (t, 4H, J = 4 Hz); 3.66 (q, 4H, J = 8 Hz); 4.89 (t, 2H, J = 8 Hz); 6.69 (s, 1H); 11.67 (s, 1H) ; ^{13}C-NMR (DMSO-d_6 75 MHz): δ 17.29; 27.07; 39.17; 54.99; 58.58; 101.08; 121.05; 142.17; 143.87; 156.12; 169.17; 176.92.

N-(2'-((2-hydroxy-2-methylpropyl)amino)-4-methyl-[5,5'-bithiazol]-2-yl)pivalamide 4c: Yield: 96%; MS (ESI) $[M+H]^+$ = 369,20 m/z; ^{1}H-NMR (DMSO-d_6 300 MHz): δ 1.14 (s, 6H); 1.23 (s, 9H); 2.44 (s, 3H); 3.23 (d, 2H, J = 4 Hz); 4.59 (s, 1H); 6.56 (s, 1H); 7.61 (t, 1H, J = 4 Hz); 11.64 (s, 1H); ^{13}C-NMR (DMSO-d_6, 75 MHz): δ 17.35; 27.08; 27.88; 56.31; 70.06; 100.65; 121.23; 142.42; 142.92; 155.99; 169.06; 176.86.

N-(2'-(cyclohexyl(2-hydroxy-2-methylpropyl)amino)-4-methyl-[5,5'-bithiazol]-2-yl)pivalamide 4d: Yield: 20%; MS (ESI) $[M+H]^+$ = 451,31 m/z; ^{1}H-NMR (MeOD 400 MHz): δ 1.24 (s, 6H); 1.31 (s, 9H); 1.41-1.45 (m, 3H); 1.67-1.70 (m, 3H); 1.88-1.91 (m, 4H); 2.47 (s, 3H); 3.33-3.41 (m, 1H); 3.49 (s, 2H); 6.65 (s, 1H); ^{13}C-NMR (MeOD 100 MHz): δ 15.63; 25.03; 25.75; 25.89; 25.92; 26.85; 29.81; 38.74; 57.72; 64.30; 71.09; 74.52; 101.71; 120.74; 142.56; 142.88; 171.91.

Water Solubility

Each solid compound (1.0 mg) was added to 1.0 mL of buffer solution at pH 7.4 (25 mM Hepes, 140 mM NaCl). Each sample was mixed at 20 °C, in a shaker water bath for 24 h. The resulting suspension was filtered through a 0.45 µm nylon filter (Acrodisc). The concentration of each compound in solution was determined by UV/LC-MS or LC-MS/MS by comparison with the

appropriate calibration curve. For each compound, the determination was performed in three independent experiments.

Parallel Artificial Membrane Permeability Assay (PAMPA)

Each 'donor solution' was prepared from a solution of each compound (DMSO, 1 mM) diluted with 25 mM Phosphate Buffered Saline (PBS) at pH 7.4, up to a final concentration of 500 µM. The donor wells were filled with 150 µL of 'donor solution'. The filters were coated with 5 µL of a solution of phosphatidylcholine in dodecane 1% (w/v) and the lower wells filled with 300 µL of 'acceptor solution' (50% v/v DMSO and PBS). The sandwich plate was assembled and incubated for 5 hours at room temperature under gentle shaking. At the end of the incubation time, the sandwich was disassembled and the amount of compound in the donor and acceptor wells was measured by LC-UV-MS. For each compound, the determination was performed in three independent experiments.

Apparent permeability (P_{app}) and membrane retention (%MR) were calculated according to the equations reported in reference [1].

Stability tests

Stability in Human Plasma

Pooled human plasma (1.5 mL, 55.7 µg protein/mL) [2], PBS (1.4 mL, pH 7.4, 25 mM) and tested compound dissolved in DMSO (100 µL, 3.0 mM) were mixed in a test tube that was incubated at 37 °C. At set time points (0.0, 0.08, 0.25, 0.50, 1.0, 3.0, and 24.0 h), samples of 150 µL were taken, mixed with 600 µL of cold acetonitrile and centrifuged at 5000 rpm for 15 min [3]. The supernatant was removed and analyzed by LC-UV-MS. For each compound, the determination was performed in three independent experiments.

Stability in Medium/Serum solution

The stability of compounds under the experimental conditions used in the ENTRY-DYRA antiviral assay was evaluated. Each tested compound dissolved din DMSO (1.0 mL, 400 µM) was mixed in a test tube with EMEM, 2 mM L-glut, 1% FBS, 1% Pen/Strep solution (final concentration of the compound of 200 µM) and then incubated at 37 °C. At set time points (0.0, 0.08, 0.25, 0.50, 1.0, 3.0, and 24.0 h), samples of 100 µL were taken, mixed with 400 µL of cold acetonitrile and centrifuged at 5000 rpm for 15 min. The supernatant was removed and analyzed by UV/LC-MS. For each compound, the determination was performed in three independent experiments.

Biology

Cells and viruses for ZIKV/SARS-CoV-2 testing

The H/PF/2013 ZIKV strain belonging to the Asian lineage was kindly provided by the Istituto Superiore di Sanità (Rome, Italy) and SARS-CoV-2 strain, belonging to lineage B.1 (EPI_ISL_2472896) was kindly provided by the Department of Biomedical and Clinical Sciences Luigi Sacco, University of Milan. [4] VERO E6 cell line (ATCC® CRL-1586) was used to propagate and titrate both viral strains. Huh7 (kindly provided by Istituto Toscano Tumori, Core Research Laboratory, Siena, Italy) and Calu-3 (ATCC® HTB-55) cell lines were used to determine the antiviral activity of candidate compounds. VERO E6 and Huh7 were maintained in high glucose Dulbecco's Modified Eagle's Medium with sodium pyruvate and L-glutamine (DMEM; Euroclone), Calu-3 in Minimum Essential Medium Eagle (EMEM; Euroclone) supplemented with 2 mM L-glutamine (L-glut, Euroclone). Both culture media were supplemented with 10% Fetal Bovine Serum (FBS; Euroclone) and 1% Penicillin/Streptomycin (Pen/Strep, Euroclone). The same medium with a lower FBS concentration (1%) was used for the viral propagation and drug susceptibility testing. Cells were incubated at 37°C in a humidified incubator supplemented with 5% CO_2. All the viral stocks were titrated by plaque reduction assay (PRA) as previously described. [5]

Drugs and cytotoxicity assay

Cytotoxicity of investigational compounds was determined by CellTiter-Glo 2.0 Luminescent Cell Viability Assay (Promega) according to the manufacturer’s protocol. The luminescence values obtained from cells treated with investigational compounds or DMSO control were measured through the GloMax® Discover Multimode Microplate Reader (Promega) and elaborated with the GraphPad PRISM software version 6.01 (La Jolla) to calculate the half-maximal cytotoxic concentration (CC_{50}). Sofosbuvir (cat. HY-15005) and camostat mesylate (cat. HY-13512) were purchased from MedChem Express (https://www.medchemexpress.com) and resuspended in 100% DMSO and water, respectively. Once determined the CC_{50}, for each compound was chosen a not-toxic dose used as starting drug concentration in the subsequent antiviral assays.

Antiviral assays (ZIKV, SARS-CoV-2)

To determine the antiviral activity of candidate compounds against ZIKV and SARS-CoV-2, a direct yield reduction assay (DYRA) based on infection of cells in presence of serial drug dilutions was performed as previously described. [6,7] Briefly, 7,000 Huh7 cells per well were infected with 50 $TCID_{50}$ of ZIKV in 96-well plates for 1 h at 37°C with 5% CO_2 and after virus removal, serial dilutions of each tested compound, starting from the not-toxic dose, were added to the cells and incubated at 37°C with 5% CO_2. Similarly, 10,000 Calu-3 were infected with 100 $TCID_{50}$ of SARS-CoV-2 in 96-well plates for 2 h at 37°C with 5% CO_2 and after virus removal, serial dilutions of each tested

compound were added to the cells and incubated at 37°C with 5% CO_2. After 72 h of incubation, the antiviral activity was measured on cell monolayer by immunodetection assay (IA). [6,7] The IA protocol was adapted for the detection of SARS nucleocapsid protein in infected Calu-3 cells. Briefly, fixation and permeabilization were performed as previously described and cell lines were incubated for 1 h with a monoclonal SARS Nucleocapsid Protein Antibody (Novus, cat. AP201054) diluted 1:1000 in blocking buffer (PBS containing 1% BSA and 0.1% Tween 20). After washing, monolayers were incubated for 1 h with a polyclonal HRP-coupled anti-mouse IgG secondary antibody (Novus Bio NB7570) diluted 1: 5,000 in blocking buffer. After cell washing, the 3,3′,5,5′-Tetramethylbenzidine substrate (Sigma Aldrich) was added to each well and the reaction was stopped with one volume of 0.5 M sulfuric acid. Absorbance was measured at 450 nm optical density (OD450) using the Absorbance Module of the GloMax® Discover Multimode Microplate Reader (Promega).

Yield reduction protocol was adapted to evaluate the anti-SARS-CoV-2 activity of investigational compounds as entry inhibitor (ENTRY-DYRA). To this purpose, 10,000 Calu-3 in 96-well plates were treated for 1 h with serial dilutions of drugs and at the end of incubation infected with 100 $TCID_{50}$ of SARS-CoV-2 at 37°C with 5% CO_2. After 2 hours of adsorption, compound and virus were removed and replaced with fresh infection medium. After 72h of incubation, the antiviral activity was measured on cell monolayer by IA.

The half-maximal inhibitory concentration (IC_{50}) was calculated through a non-linear regression analysis of the dose-response curves generated with GraphPad PRISM software version 6.01. In each test, sofosbuvir and camostat mesylate were used as reference compounds against flaviviruses and SARS-CoV-2, respectively. Infected and uninfected cells without drugs were used to calculate the 100% and 0% of viral replication, respectively. Selectivity Index (SI) was calculated as ratio between CC_{50} and IC_{50}.

Multicycle CPE reduction antiviral assay (HRV-02, HRV-14)

A multicycle CPE reduction assay to determine the antiviral activity of the compounds was performed as previously described in ref. [8]. In short, subconfluent HelaR19 (ATCC catalogue no. CCL2) were treated with serial dilutions of compounds and the cells were subsequently infected with virus at a MOI 0.01 for HRV-02 and HRV-14. Cells were incubated at 37°C for 3 days until full CPE was observed in virus infected untreated cell controls. In parallel, a cell viability assay to determine cytotoxicity was performed using the Aqueous One Solution Cell Proliferation Assay (Promega) according to the manufacturer’s protocol. The optical density at 490 nm was determined using a BioTek Gen5 microplate reader. The concentration of compound that inhibits virus-induced CPE by 50% (EC50) was calculated by nonlinear regression analysis with GraphPad Prism Version 6.

PI(4)P lipid staining

The staining methods was performed as previously described in (doi: 10.1042/BJ20090428) with slight modifications. HeLa R19 cells grown on coverslips in 24- well plates were treated with 10 μM of compound for 1 hour, fixed with 2% formaldehyde (FA) in PBS for 15 minutes and permeabilized with 100 μM digitonin in Buffer A (20 mM Pipes, pH 6.8, 137 mM NaCl, 2.7 mM KCl) for 10 minutes. The cells were blocked with 5% Normal Goat Serum (NGS) and 50 mM NH_4Cl in Buffer A for 45 minutes. Between each step, cells were washed three times PBS. The cells were incubated with the primary antibody mouse anti-PI4P IgM (Echelon Biosciences, Z-P004) diluted 1:100 in buffer A supplemented with 5% NGS for 1 hour on ice, and with the secondary antibody Alexa Fluor 594 Goat anti-Mouse IgM (Invitrogen) diluted 1:400 in buffer A supplemented with 5% NGS and 1:1000 DAPI solution for another hour also on ice. The cells were fixed with 2% FA in PBS for 10 minutes, washed with PBS + 50 mM NH4Cl and mounted with Fluorsave on coverslides. The slides were imaged with an Olympus BX60 fluorescence microscope.

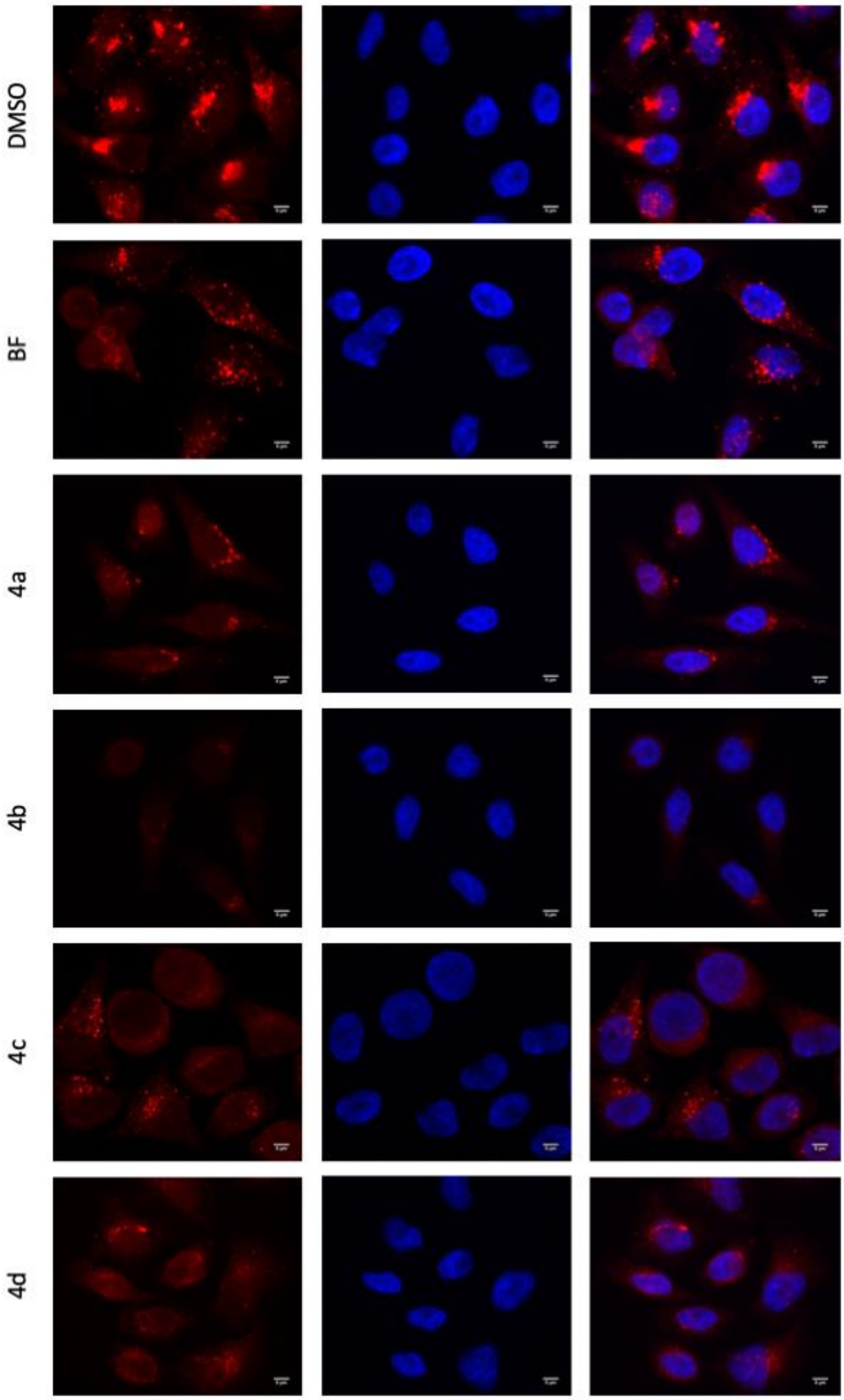

Supplementary Figure S1: Effect of compounds 4a-4d on intracellular PI4P levels. HeLa R19 cells were treated with 10 µM compound, stained for PI4P and imaged with fluorescence microscopy. As a positive control, the known PI4Kβ inhibitor BF738735 was used. [9] Examples are shown from one experiment representative of two independent experiments. Scale bars correspond to 5 µm.

Viability assays in human lymphocytes

Human peripheral blood mononuclear cells were isolated by means of Lympholyte® density gradient centrifugation starting from buffy coat. Mononuclear cells were incubated for 4h with the compounds in study at 50μM or with the vehicle (DMSO 0.5%). Following incubation with propidium iodide 10 μg/mL for 1 minute in the dark, at room temperature, mononuclear cells were immediately subjected to flow cytometry analysis: viable cells were identified as cells negative for the red fluorescence. The percentage of viability among lymphocytes, identified through the gating in the forward scatter (FSC)-side scatter (SSC) (FSC low: SSC low) plot of mononuclear cells, was calculated with respect to lymphocytes exposed to vehicle (DMSO 0.5%).

In vitro kinase inhibition assays

Recombinant full length, HIS6-tagged PI4KIIIβ was purchased from ProQinase (Germany).Recombinant full length, N-terminal FLAG-tagged full-length human PI3KR1 (p110α - GenBank® Accession Number U79143 - and human p85α - no tag; GenBank® Accession Number XM_043865) was purchased from ProQinase (Germany).

<u>Assay conditions:</u>

PI4KIIIβ and PI3KR1 reactions were performed in 10 μL using 20 mM Tris-HCl pH 7.5, 0.125 mM EGTA, 2 mM DTT, 0.04% Triton, 3 mM $MgCl_2$, 3 mM $MnCl_2$, 20 μM ATP, 0.01 μCi γ-P33 ATP, 200 μM Pi:3PS, 10% DMSO, 0.4 ng/μL of PI4KIIIβ and 7.6 ng/μL of PI3KR1. All reactions were performed at 30 °C for 10 min. Reactions were stopped by adding 5μL of phosphoric acid 0.8%. Aliquots (10 μL) were then transferred into a P30 Filtermat (PerkinElmer), washed five times with 0.5% phosphoric acid and four times with water for 5 min. The filter was dried and transferred to a sealable plastic bag, and scintillation cocktail (4 mL) was added. Spotted reactions were read in a scintillation counter (Trilux, Perkinelmer). IC_{50} values were obtained according to Equation (1), where *v* is the measured reaction velocity, *V* is the apparent maximal velocity in the absence of inhibitor, I is the inhibitor concentration, and IC_{50} is the 50% inhibitory concentration.

$$v = V/\{1+(I/IC_{50})\} \quad (1)$$

<u>Lipidic substrate preparation:</u>

PI: phosphatidylinositol (Sigma); PS: 2-Oleoyl-1-palmitoyl-sn-glycero-3-phospho-L-serine (Sigma). PI and PS were dissolved in chloroform/methanol 9:1 and mixed at a 1:3 ratio. After chloroform/methanol evaporation, water was added to 1:62.5 w/v and the mixture sonicated to clarity.

References

[1] E. Rango, et al. *European Journal of Medicinal Chemistry* **2021**, *223*, 113653, doi:10.1016/j.ejmech.2021.113653.

[2] M. Bradford, *Analytical Biochem.* **1976**, *72*, 248–254.

[3] B.L. Barthel, D.L. Rudnicki, T.P. Kirby, S.M. Colvin, D.J. Burkhart, T.H. Koch, *J. Med. Chem.* **2012**, *55*, 6595–6607.

[4] A. Lai, A. Bergna, et al. *Viruses* **2020**, *12* (8), 798.

[5] I. Vicenti, A. Boccuto, et al. *Virus Research* **2018**, *244*, 64-70.

[6] I. Vicenti, F. Dragoni, et al. *SLAS Discov.* **2020**, *25*, 506-514

[7] A. Boccuto, F. Dragoni, et al. *Int. J. Mol. Sci.* **2021**, 22(5):2670.

[8] L. Bauer, R. Manganaro, et al. *PLoS Biol.* **2020**, *18* (11), e3000904.

[9] A. M. MacLeod, D. R. Mitchell, et al. *ACS Med. Chem. Lett.* **2013**, *4* (7), 585–589.
